# Improving care quality through hybrid implementation/effectiveness studies: Best practices in design, methods, and measures

**DOI:** 10.1186/1748-5908-10-S1-A29

**Published:** 2015-08-20

**Authors:** Amy N Cohen, Alison B Hamilton, Mona Ritchie, Brian S Mittman, JoAnn E Kirchner, Gail E Wyatt, John C Fortney, Gerhard Hellemann, Honghu Liu, Geoffrey M Curran, Fiona Whelan, Alicia M Eccles, Louise E Parker, Kirk McNagny, Craig S Hutchinson, Annapurni B Teague, Christopher Reist, Alexander S Young

**Affiliations:** 1 Desert Pacific Mental Illness Research, Education, and Clinical Center (MIRECC), Greater Los Angeles VA Healthcare Center, Los Angeles, CA, 90073, USA; 2 Department of Psychiatry and Biobehavioral Sciences, University of California, Los Angeles, CA, 90095, USA; 3 Center for the Study of Healthcare Innovation, Implementation and Policy (CSHIIP), Greater Los Angeles VA Healthcare Center, Los Angeles, CA, 91343, USA; 4 Mental Health Quality Enhancement Research Initiative (QUERI), Department of Veterans Affairs, North Little Rock, AR, 72114, USA; 5 Department of Psychiatry, University of Arkansas for Medical Sciences, Little Rock, AR, 72114, USA; 6 Center for Implementation Practice and Research Support, Greater Los Angeles VA Healthcare System, Los Angeles, CA, 91343, USA; 7 Department of Research and Evaluation, Kaiser Permanente Southern California, Pasadena, CA, 91101, USA; 8 HSR&D Center of Innovation for Veteran-Centered and Value-Driven Care, VA Puget Sound Healthcare System, Seattle, WA, 98108, USA; 9 Department of Psychiatry and Behavioral Sciences, University of Washington, Seattle, WA, 98195, USA; 10 Semel Institute Statistics Core (SISTAT), Department of Psychiatry and Biobehavioral Sciences, University of California, Los Angeles, CA, 90095, USA; 11 Department of Dentistry, University of California, Los Angeles, CA, 90095, USA; 12 Department of Public Health, University of California, Los Angeles, CA, 90095, USA; 13 VA Center for Mental Healthcare & Outcomes Research, Central Arkansas VA Healthcare System, North Little Rock, AR, 72114, USA; 14 Department of Management and Marketing, University of Massachusetts, Boston, MA, 02125, USA; 15 Mental Health Care Group, VA Long Beach Healthcare System, Long Beach, CA, 90822, USA; 16 Department of Psychiatry & Human Behavior, University of California, Irvine, CA 92868, USA; 17 Office of the Chief of Staff, Michael E Debakey VA Medical Center, Houston, TX, 77030, USA; 18 Office of the Chief of Staff, VA Long Beach Healthcare System, Long Beach, CA, 90822, USA; 19 Center for Health Services, University of California, Los Angeles, CA, 90024, USA

## Panel overview abstract

Implementation research is the scientific study of methods that promote systematic uptake of research findings and other evidence-based practices into routine practice, thereby improving the quality and effectiveness of health services. As the field has progressed over the past decades, substantial advances continue in the development and application of implementation-related theories as well as innovative implementation strategies and methods.

This session will provide three examples of implementation research studies that are designed to improve care through the use of evidence. The examples come from three health services areas: primary care, mental health, and HIV; from funded NIH and VA studies; and from community and hospital settings in rural and urban sites. Across the three examples, six different theories were used-providing ample examples of linking conceptual models and frameworks to study design, implementation strategies, and measurements. Each presentation will highlight the ways in which the conceptual theory was configured into the overall research design and evaluation, as well as common challenges and lessons learned while conducting implementation research.

Blended facilitation to enhance PCMH program implementation: conceptual, design, and measurement considerations

Integrated primary care mental health evidence-based programs improve care. The Department of Veterans Affairs mandated and provided limited national level implementation support for Primary Care - Mental Health Integration (PC-MHI) but VA facilities were slow to implement them. The Blended Facilitation study was funded by the VA to implement and evaluate an innovative implementation facilitation (IF) strategy that included a national external expert facilitator with expertise in implementation science and PC-MHI who mentored and worked with two internal regional facilitators to help clinics implement PC-MHI.

According to the PARIHS framework, successful implementation is a function of the dynamic interaction between evidence, context and facilitation. This presentation will describe how PARIHS guided the application of the IF strategy and the study's design. For this quasi-experimental, Hybrid Type 3 study, we used mixed methods to test the effectiveness of the IF strategy and assess organizational context, perceptions of evidence, and facilitation activities. We used a consensus matching approach to select sixteen clinics that were unlikely to implement PC-MHI without assistance across four VA regions. The RE-AIM framework guided our test of IF's effectiveness using administrative data and program component interviews to measure RE-AIM dimensions. We conducted monthly debriefing interviews with and collected time data from facilitators and measured site level contextual and evidence factors through key informant interviews. We also conducted intensive case studies at four IF clinics to assess stakeholders' perception of IF's processes and value.

There are limited examples of Hybrid Type 3 studies and this is an excellent large-scale example of one that also details how the conceptual frameworks guide design, strategies, and measurement. This presentation will discuss the strengths and weaknesses of the conceptual framework, the IF strategy and the study design we selected and lessons we learned about the challenges of conducting implementation research within the context of a VA policy initiative.

Implementing high priority evidence-based practices for individuals with schizophrenia: conceptual, design, and measurement considerations

Most individuals with schizophrenia receive about half of the indicated treatments that have been shown to improve quality of life and health. As a result, outcomes in routine practice are much worse than in state-of-the-art care. This gap in implementation- both in delivery and uptake of services-was addressed in the VA HSR&D QUERI-funded EQUIP study.

This Hybrid Type 2 study integrated two conceptual frameworks. The Simpson Transfer Model (STM) is a program change model that examines readiness to change at the organizational and provider levels and posits that research moves into practice through 5 phases: exposure, adoption, implementation, practice, and sustainability. Since the STM does not recommend specific behavior change tools, the PRECEDE model guided the choice of a multifaceted implementation strategy to influence the adoption of behavior changes necessary for implementation success and positive patient outcomes.

Across four states, eight specialty mental health programs were assigned to implementation or usual care. Two evidence-based services were targeted: Wellness and Supported Employment. Veterans with schizophrenia (n = 801) and clinicians (n = 201) were enrolled. At implementation sites, organizational readiness data were used to tailor implementation. The implementation strategy included patient-facing kiosks for routine assessment, evidence-based quality improvement methods, social marketing, opinion leaders, provider and patient education, and continual feedback to staff. Mixed methods were used to evaluate implementation and effectiveness. Patients and clinicians were surveyed and interviewed at baseline and 15 months. Intervention clinicians were also interviewed mid-study. A cost effectiveness evaluation was included.

This presentation will discuss the key decisions made in selecting the conceptual frameworks and how those decisions impacted the study design including preparation and implementation, measure choice, and assessment timing. This is one of the first studies to improve care quality in specialty mental health in diverse VA settings and therefore provides an excellent example for the application of integrated conceptual frameworks and a multifaceted implementation strategy.

Implementation and effectiveness of an evidence-based intervention in community-based organizations: conceptual, design, and measurement considerations

The HIV/AIDS epidemic continues to disproportionately affect African-American communities in the US, particularly those located in urban, resource-constrained areas. This five-year Hybrid Type 2 study investigates community-based implementation, effectiveness, and sustainability of 'Eban II,' an evidence-based risk reduction intervention for African-American heterosexual, serodiscordant couples.

Key decisions in the design of this study included how to balance investigating implementation and effectiveness (and resources associated with each), which models/theories to apply in order to guide organization-level implementation and couples-level behavioral change, which implementation strategies to select for optimal uptake, how to measure implementation, how to analyze organizational-level data, how to capture implementation costs, and how to conceptualize and measure sustainability. Each of these decisions will be described in this presentation. Specifically, we will describe our: hybrid design and dynamic waitlisted effectiveness study, application of the Program Change Model (PCM, a phased model of organizational change) and social cognitive theory, selection of implementation strategies and tools for each phase of the PCM, quantitative and qualitative implementation and effectiveness measures/instruments, multi-level hierarchical modeling with a multi-level nested structure analysis, measures of implementation costs and potential cost savings, and approach to sustainability as defined by research-independent delivery of the intervention and relative use of technical assistance.

This study provides an excellent example of a funded study using a hybrid design in resource-constrained community settings. The study provides an illustration of the value of community supports and expertise throughout the study to shape the design, implementation strategies, and measurements. The impact of the organizational feedback and contingencies will be briefly articulated with regard to lessons learned for future community-based implementation studies.
